# Surgical treatment and overall survival in patients with right-sided obstructing colon cancer—a nationwide retrospective cohort study

**DOI:** 10.1007/s00384-023-04541-3

**Published:** 2023-10-05

**Authors:** Jeske R. E. Boeding, Marloes A. G. Elferink, Pieter J. Tanis, Johannes H. W. de Wilt, Paul D. Gobardhan, Cornelis Verhoef, Jennifer M. J. Schreinemakers

**Affiliations:** 1 Department of Surgery, Amphia Hospital, Breda, The Netherlands; 2 Department of Surgical Oncology, Erasmus MC Cancer Institute, Rotterdam, The Netherlands; 3 Department of Research & Development, Netherlands Comprehensive Cancer Organisation, Utrecht, The Netherlands; 4 Department of Surgery, Amsterdam UMC, University of Amsterdam, Amsterdam, The Netherlands; 5 Department of Surgery, Radboud University Medical Centre, Nijmegen, The Netherlands

**Keywords:** Obstruction, Colon cancer, Survival, Treatment, Mortality, Right-sided

## Abstract

**Purpose:**

The aim of this study was to compare baseline characteristics, 90-day mortality and overall survival (OS) between patients with obstructing and non-obstructing right-sided colon cancer at a national level.

**Methods:**

All patients who underwent resection for right-sided colon cancer between January 2015 and December 2016 were selected from the Netherlands Cancer Registry and stratified for obstruction. Primary outcome was 5-year OS after excluding 90-day mortality as assessed by the Kaplan-Meier and multivariable Cox regression analysis.

**Results:**

A total of 525 patients (7%) with obstructing and 6891 patients (93%) with non-obstructing right-sided colon cancer were included. Patients with right-sided obstructing colon cancer (OCC) were older and had more often transverse tumour location, and the pathological T and N stage was more advanced than in those without obstruction (*p* < 0.001). The 90-day mortality in patients with right-sided OCC was higher compared to that in patients with non-obstructing colon cancer: 10% versus 3%, respectively (*p* < 0.001). The 5-year OS of those surviving 90 days postoperatively was 42% in patients with OCC versus 73% in patients with non-obstructing colon cancer, respectively (*p* < 0.001). Worse 5-year OS was found in patients with right-sided OCC for all stages. Obstruction was an independent risk factor for decreased OS in right-sided colon cancer (HR 1.79, 95% CI 1.57–2.03).

**Conclusion:**

In addition to increased risk of postoperative mortality, a stage-independent worse 5-year OS after excluding 90-day mortality was found in patients with right-sided OCC compared to patients without obstruction.

**Supplementary Information:**

The online version contains supplementary material available at 10.1007/s00384-023-04541-3.

## Introduction

Emergency resection with primary anastomosis is still the mostly performed surgical option in patients with right-sided obstructing colon cancer (OCC) [1–3]. However, evidence for emergency resection with primary anastomosis is mainly based on everyday clinical practice and guideline recommendations are based on low quality evidence [4–6].

The current literature for right-sided OCC shows high morbidity and mortality rates after emergency surgery, compared to elective surgery in patient with non-obstructing colon cancer [7–9]. Alternative (staged) treatment options have been proposed over the years, such as stent placement and decompressing ileostomy followed by tumour resection [10–14]. These alternative treatment options, avoiding emergency resection in patients with obstruction, have mainly been analysed for left-sided OCC [4, 15, 16]. For right-sided OCC, studies comparing different surgical and non-surgical treatment options are scarce [10, 17].

The main focus in the literature on OCC has been on short-term outcomes. Although emergency setting has been reported to be associated with worse overall survival (OS), the independent contribution of obstruction to long-term prognosis has been less well analysed [18, 19]. Identified differences in OS between obstructing and non-obstructing tumours might predominantly be caused by the increased mortality after emergency treatment, while it is unclear whether obstruction is still a prognostic factor if analysing survival beyond the 90-day postoperative period.

The aim of this study was to compare baseline characteristics, 90-day mortality, and 5-year OS after excluding 90-day mortality, between patients with obstructing and non-obstructing right-sided colon cancer patients at a national level. In addition we aim to determine whether obstruction is an independent predictor for OS in patients with right-sided colon cancer.

## Materials and methods

### Study design and population

This is a nationwide retrospective cohort study containing all patients, diagnosed with right-sided colon cancer between January 2015 and December 2016, who were treated with surgical resection. The data were collected from the Netherlands Cancer Registry (NCR). The NCR comprises data on all newly diagnosed malignancies and is hosted by the Netherlands Comprehensive Cancer Organisation (IKNL). Topography and morphology are coded using the International Classification of Diseases for Oncology [20]. Data were obtained after approval of the study protocol by the Dutch PLCRC (Prospective National (‘Landelijk’) Colorectal Cancer Cohort) review board.

All patients with histologically proven right-sided colon carcinoma (e.g. caecum, ascending colon, hepatic flexure, transverse colon proximal to the splenic flexure (C18.0–C18.2–C18.3–C18.4), aged 18 years or older who underwent surgical resection were included. After patient selection by IKNL, an anonymised database was made available for analyses. Exclusion criteria were presentation with bowel perforation, two synchronous primary colon tumours treated by two surgical resections, missing information about the presence or absence of obstruction or bowel perforation.

### Data extraction and definitions

The following data were collected: patient characteristics (age, sex, American Society of Anaesthesiologists (ASA) score) tumour characteristics (clinical and pathological tumour stage, primary tumour location, obstruction (ileus), tumour morphology), diagnostic characteristics (complete imaging of the colon preoperatively), and surgical characteristics (urgency of surgery, resection type, completeness of resection, per- or postoperative stoma creation).

Tumour-Node-Metastasis (TNM) staging model, 7^th^ edition (TNM 7), was used [21]. Tumour stage was classified following Union for International Cancer Control (UICC). Stage was derived from the pathological TNM (pTNM) information. If the pTNM was missing the clinical TNM (cTNM) was used to determine the tumour stage. Stage IV disease was defined as the detection of metastases before the start of treatment or during surgical exploration. The urgency of surgical intervention was defined as follows: acute surgery (surgery scheduled less than 12 h in advance), urgent surgery (surgery planned at least 12 h in advance, no elective surgery), elective surgery or staged surgery (including bridging strategy using stent placement or decompressing stoma). Emergency surgery included both acute and urgent surgery. Completeness of resection was defined as resection with a resection margin > 1 mm. In case of incomplete resection, distinction was made between microscopic incomplete resection (resection margin ≤ 1 mm) and macroscopic incomplete resection (the surgeon indicates that tumour was left behind).

Survival time could be calculated due to annual linkage with data from the Municipal Personal Records Database, containing information on vital status and date of death from all Dutch inhabitants. Follow-up time was calculated as the time between resection and death or last time of follow-up (1st February 2022). Postoperative mortality was defined as death within 90 days after surgery. In case of postoperative mortality, patients were excluded for OS analysis. Sub-analyses of OS were performed for stage I–II, stage III and stage IV disease. Among patients with obstruction, sub-analyses of OS were performed comparing bridge to elective resection and emergency resection.

### Outcome parameters

The primary outcome measure was 5-year OS in the population surviving 90 days postoperatively. Additional study outcome of this study included 90-day mortality.

### Statistical analysis

Statistical analysis was performed using IBM SPSS Statistics Program version 25 and R-studio version 1.4.1717. Baseline characteristics were evaluated using descriptive statistics. Continuous variables were described as median with interquartile range (IQR). Categorical variables were described as counts and percentages. Fisher’s exact test or the *χ*^2^ test was used for data analysis of categorical variables. Kaplan–Meier curves were constructed to estimate 5-year OS of patients with or without obstruction and compared using the log-rank test. The Independent-Samples Median test was performed to determine statistical differences between median follow-up time of both groups. Uni- and multivariable Cox proportional hazard regression models were fitted for OS to determine whether obstruction was an independent predictor. Results from the proportional hazard regression analyses were reported as Hazard Ratio (HR) with corresponding 95% confidence interval (CI). Statistical significance level was set at an *α* of 0.05.

### Ethical standard

The Medical Research Ethics Committees United (MEC-U) was consulted for ethical approval. They confirmed that, for this study (reference number W20.135), the medical research involving Human Subject Act (WMO) does not apply.

## Results

**Fig. 1:**
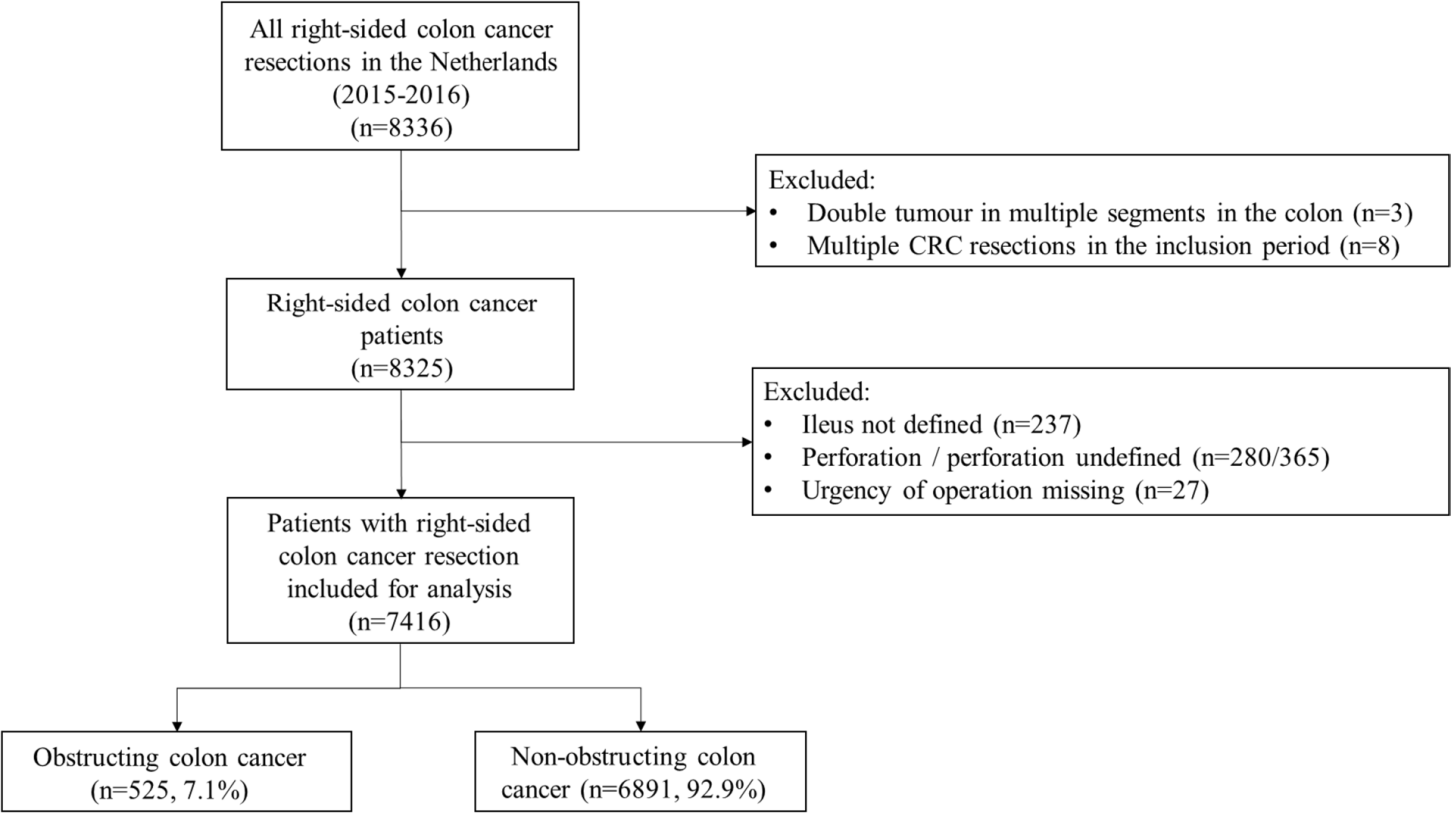
Flowchart of inclusion

In total 7416 patients with right-sided colon cancer treated with surgical resection were included for analysis, of whom 525 patients (7%) presented with signs of obstruction (Fig. 1). The median age of all patients was 71 years (IQR 65–78), and the tumour was most often located in the caecum (*n* = 2754, 37%) (Table 1).

**Table 1:** Tumour characteristics of obstructing versus non-obstructing colon cancer

	Non-obstructing colon cancer (*n* = 6891)	Obstructing colon cancer (*n* = 525)
Year of diagnosis		
*2015*	3382 (49)	301 (57)
*2016*	3509 (51)	224 (43)
*Sex*		
*Male*	321 (47)	262 (50)
*Female*	3680 (53)	263 (50)
Age (median, IQR)	71 (65–78)	73 (65–81)
Primary tumour location		
*Caecum*	2572 (37)	182 (35)
*Ascending colon*	2504 (36)	136 (26)
*Hepatic flexure*	774 (11)	72 (14)
*Transverse colon*	1041 (15)	135 (26)
Pathological T stage		
(y)*pT0*	8 (0)	1 (0)
(y)*pT1*	647 (9)	–
*(y)pT2*	1284 (19)	18 (3)
*(y)pT3*	3978 (58)	308 (59)
*(y)pT4*	951 (14)	197 (38)
*pTx*	23 (0)	1 (0)
Pathological N stage		
*(y)pN0*	4461 (65)	183 (35)
(y)*pN1*	1491 (22)	155 (30)
(y)*pN2*	929 (13)	186 (35)
*pNx*	10 (0)	1 (0)
Tumour stage (UICC)		
*Stage I*	1656 (24)	13 (2)
*Stage II*	2678 (39)	155 (30)
*Stage III*	1973 (29)	206 (39)
*Stage IV*	560 (8)	150 (29)
*Stage X*	24 (0)	1 (0)

### Obstructing versus non-obstructing right-sided colon cancer

Patients with obstruction were significantly older compared to patients with non-obstructing right-sided colon cancer (*p* = 0.046). In right-sided OCC, the primary tumour location was most often found in the caecum. Non-obstructing colon cancer was more often found in the ascending colon, compared to OCC which was found more often in the transverse colon (*p* < 0.001). In case of OCC, patients had higher pT and pN stage compared to patients with non-obstructing colon cancer (*p* < 0.001). Along with a higher pT and pN stage, stage IV disease was found in 29% of the patients compared to 8% in patients with non-obstructing right-sided colon cancer (*p* < 0.001) (Table 1).

### Management

Acute/urgent resection was performed in 88% of the patients with right-sided OCC, compared to 2% in patients with non-obstructing colon cancer (*p* < 0.001). Staged resection was performed in 26 patients with OCC (5%). Diverting ileostomy was created more often (25% versus 7%, *p* < 0.001), and the completeness of resection was significantly lower in patients with OCC (90% versus 97%, *p* < 0.001). Postoperative mortality was significantly higher in the OCC group (10% versus 3%, *p* < 0.001) (Table 2).

**Table 2:** Surgical characteristics of obstructing versus non-obstructing colon cancer

	Non-obstructing colon cancer (*n* = 6891)	Obstructing colon cancer (*n* = 525)	*p* value
ASA classification			**< 0.001**
*ASA I*	842 (12)	44 (8)	
*ASA II*	3889 (56)	227 (43)	
*ASA III*	1675 (24)	132 (25)	
*ASA IV*	94 (1)	14 (3)	
*ASA V*	–	–	
**Missing	391 (6)	108 (21)	
Urgency of surgery			**< 0.001**
*Acute surgery*	77 (1)	262 (50)	
*Urgent surgery*	99 (1)	201 (38)	
*Elective surgery*	6715 (97)	36 (7)	
*Staged surgery*	–	26 (5)	
*Stent as bridge to surgery*	–	*3 (1)*	
*Stoma as bridge to surgery*	–	*22 (4)*	
**Missing	–	–	
Ileostomy			**< 0.001**
*None*	6435 (93)	372 (71)	
*Preoperative*	12 (0)	22 (4)	
*Peri-/postoperative*	444 (7)	131 (25)	
Resection			
*(Extended) right hemicolectomy*	6051 (88)	432 (82)	**0.001**
*Transverse resection*	268 (4)	29 (6)	
*(Extended) left hemicolectomy*	281 (4)	31 (6)	
*Subtotal colectomy*	139 (2)	8 (2)	
*Ileocecal resection*	69 (1)	14 (3)	
*Multisegment resection*	60 (1)	9 (2)	
*Other*	23 (0)	2 (0)	
Completeness of resection			**< 0.001**
*Complete margins (R0)*	6687 (97)	470 (90)	
*Incomplete margins (microscopic, R1)*	100 (2)	26 (5)	
*Incomplete margins (macroscopic, R2)*	43 (1)	16 (3)	
**Missing	54 (1)	13 (2)	
Chemotherapy			**< 0.001**
*No chemotherapy*	5198 (75)	310 (59)	
*Only neo-adjuvant chemotherapy*	80 (1)	4 (1)	
*Only adjuvant chemotherapy*	1583 (23)	206 (39)	
*Neo- and adjuvant chemotherapy*	30 (0)	5 (1)	
90-day mortality	214 (3)	52 (10)	**< 0.001**

Values in bold indicate statistical significance

*NA* not applicable

### Overall survival

**Fig. 2:**
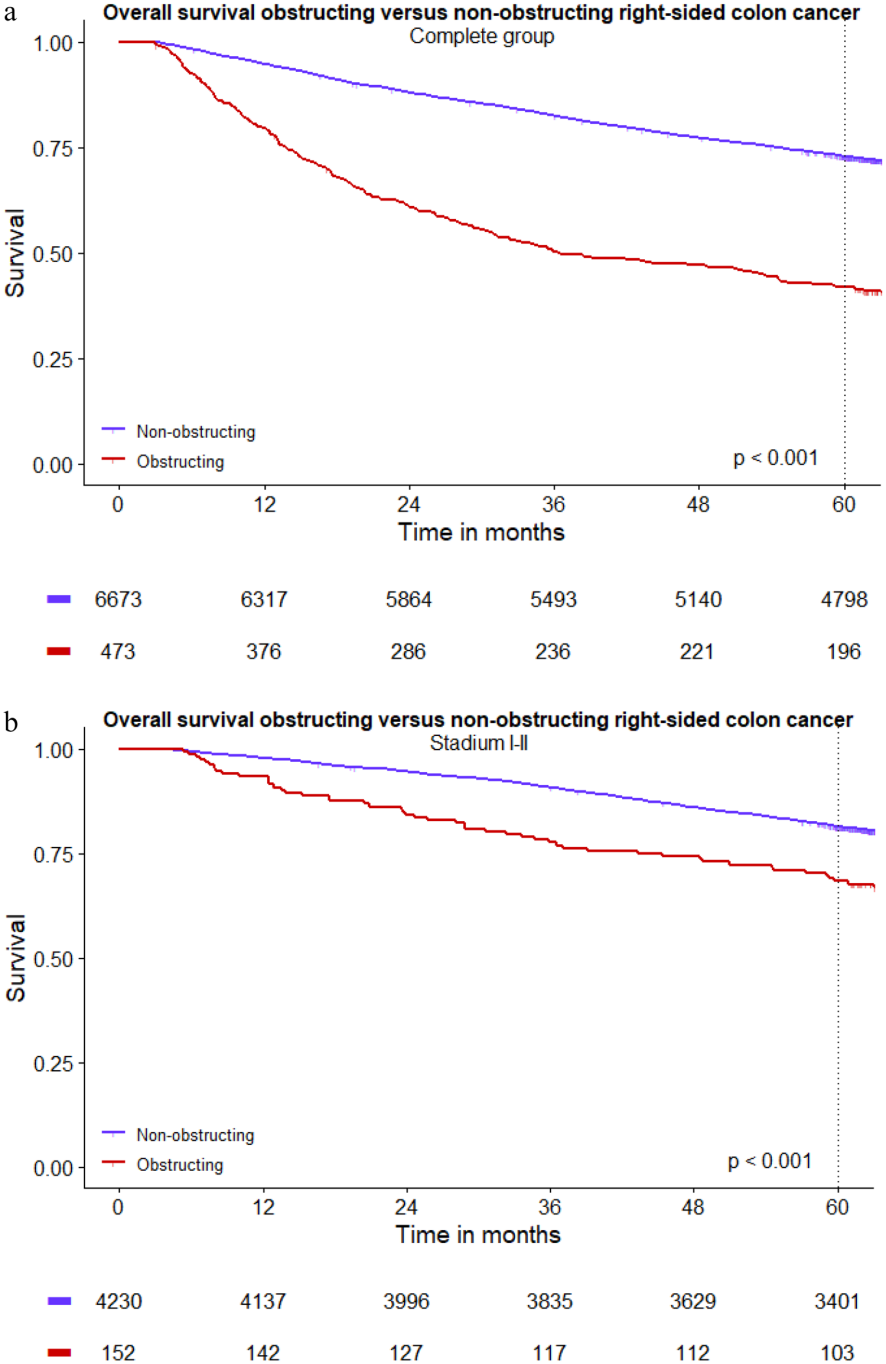

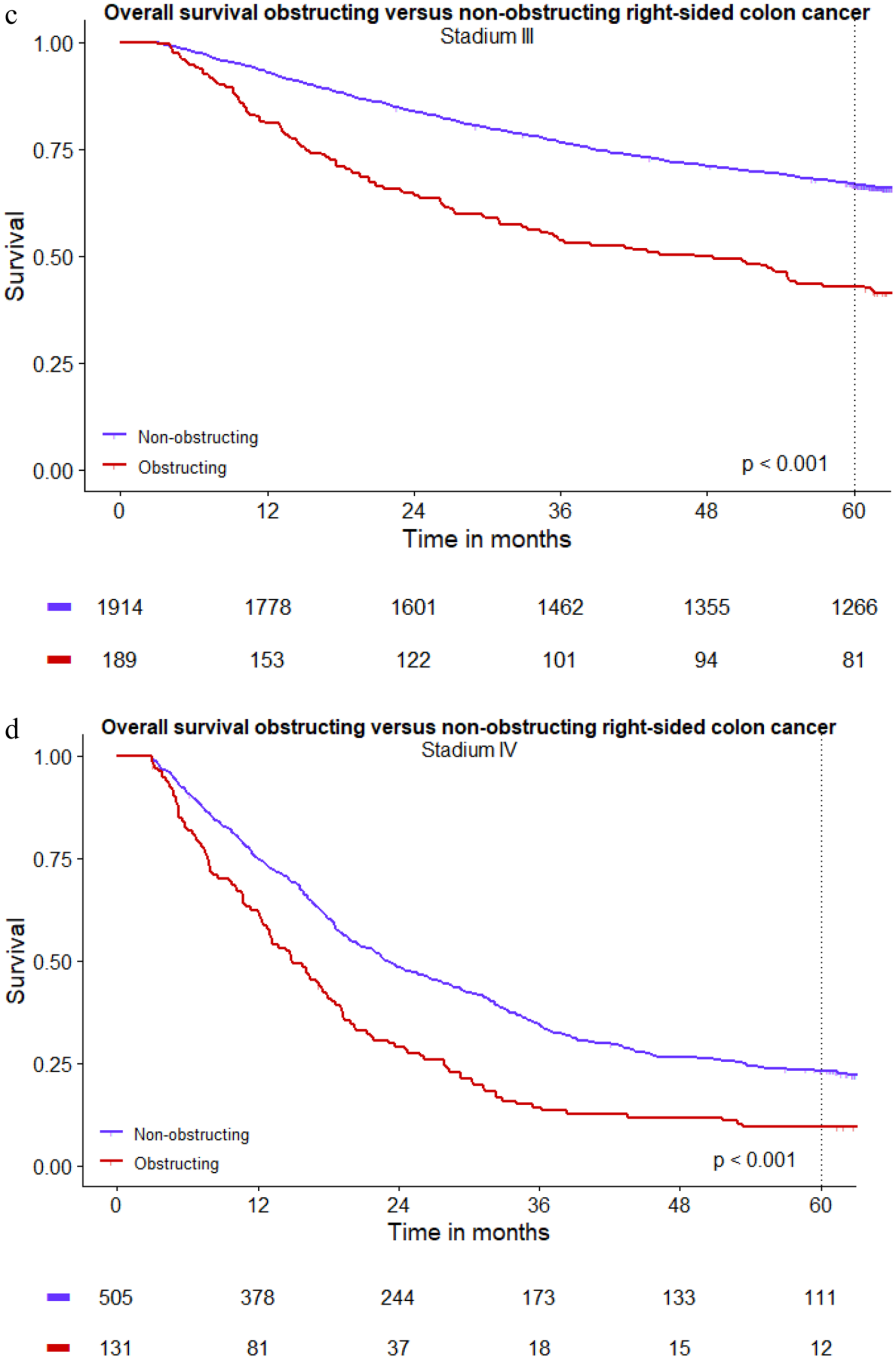
**a**–**d** Long-term overall survival obstructing versus non-obstructing right-sided colon cancer (*t*0 = date of surgery, postoperative mortality within 90 days was excluded for OS analyses)

After excluding patients who died within 90 days, a total of 7146 patients were included for OS analyses. Median follow-up time for the complete group was 5.6 years (IQR 4.0–6.2). Five-year OS showed a significant difference between obstructing (42%) and non-obstructing right-sided colon cancer (73%) (*p* < 0.001). Stratified analyses showed a worse OS in patients with obstructing colon cancer compared to patients with non-obstructing colon cancer for all stages: stage I–II disease 68% versus 81%, stage III disease 43% versus 67%, stage IV disease 9% versus 23% (*p* < 0.001) (Fig. 2a–d). After correction for sex, age, tumour location, pT stage, pN stage, pM stage, and completeness of the resection, obstruction was found to be independently associated with decreased OS in right-sided colon cancer (HR 1.79, 95% CI 1.57–2.03) (Table 3).

**Table 3:** Uni- and multivariable analysis for overall survival in all patients treated surgically for right-sided colon cancer in the Netherlands (Cox proportional hazard regression)

		Univariable analysis		Multivariable analysis	
		HR (95% CI)	*p* value	HR (95% CI)	*p* value
Sex	Male	1 (reference)		1 (reference)	
	**Female**	**0.884 (0.816**–**0.958)**	**0.003**	**0.869 (0.800**–**0.943)**	**0.001**
**Age**		**1.041 (1.036**–**1.045)**	** < 0.001**	**1.052 (1.047**–**1.057)**	**< 0.001**
Tumour location	Caecum	1 (reference)		1 (reference)	
	Ascending colon	0.912 (0.830–1.002)	0.055	0.994 (0.903–1.093)	0.897
	Hepatic flexure	1.052 (0.923–1.199)	0.444	**1.188 (1.040**–**1.357)**	**0.011**
	**Transverse colon**	**0.884 (0.781**–**0.999)**	**0.049**	0.939 (0.827–1.065)	0.326
pT	pT1-2	1 (reference)		1 (reference)	
	**pT3-4**	**2.250 (2.016**–**2.511)**	** < 0.001**	**1.366 (1.215**–**1.535)**	**< 0.001**
pN	pN0	1 (reference)		1 (reference)	
	**pN+ **	**2.516 (2.321**–**2.727)**	** < 0.001**	**1.834 (1.675**–**2.007)**	**< 0.001**
M	M0	1 (reference)		1 (reference)	
	**M+ **	**6.091 (5.517**–**6.724)**	** < 0.001**	**4.760 (4.254**–**5.326)**	**< 0.001**
Completeness of resection	R0	1 (reference)		1 (reference)	
	**R1/2**	**3.502 (2.873**–**4.268)**	** < 0.001**	**1.651 (1.347**–**2.023)**	**< 0.001**
Obstruction	No	1 (reference)		1 (reference)	
	**Yes**	**2.787 (2.465**–**3.152)**	** < 0.001**	**1.786 (1.569**–**2.034)**	**< 0.001**

Patients with mortality ≤ 90 days were excluded for this overall survival analysis. Values in bold indicate statistical significance

### Staged resection

**Fig. 3:**
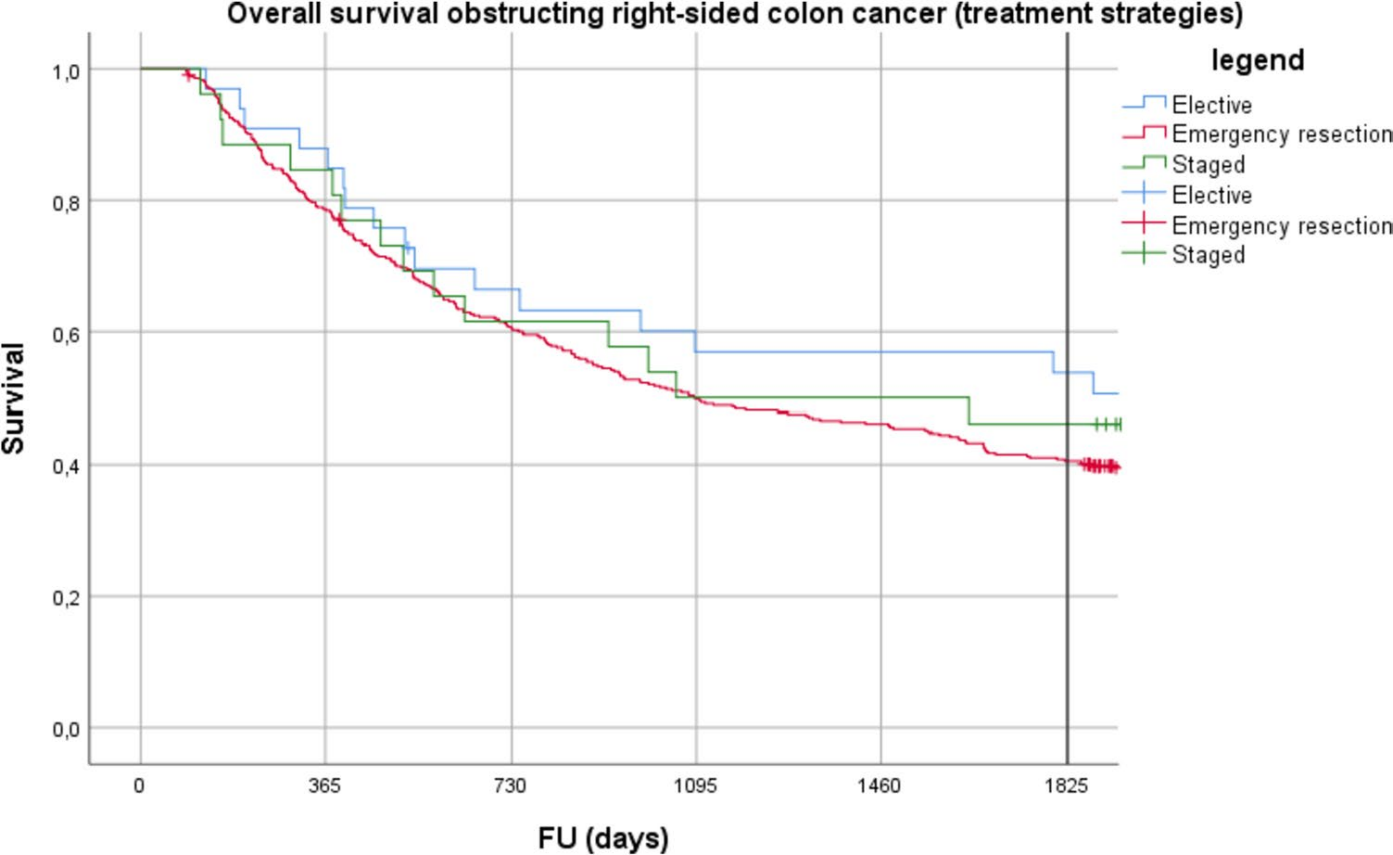
Overall survival in obstructing right-sided colon cancer staged versus non-staged treatment. Log-rank: p = 0.498

A bridge to surgery strategy using either stent or stoma was performed in 26 patients with right-sided OCC (5%). These patients were treated with preoperative stent (*n* = 3) or stoma (*n* = 22) for initial colonic decompression. No 90-day mortality was found after staged tumour resection following initial stent or stoma, compared to 11% in the emergency resection group (*p* = 0.095) (Table 4). Comparison of overall survival after exclusion of postoperative 90-day mortality revealed no significant differences between patients treated with elective resection, staged resection or emergency resection (*p* = 0.498) (Fig. 3/Table 4).

**Table 4:** Treatment strategies in right-sided obstructing colon cancer

	Emergency surgery (*n* = 463)	Staged surgery (*n* = 26)	Elective surgery (*n* = 36)
Clinical T stage			
* cT0*	1 (0)	–	1 (3)
* cT1*	1 (0)	–	1 (3)
* cT2*	14 (3)	3 (12)	3 (8)
* cT3*	88 (19)	7 (27)	7 (19)
* cT4*	62 (13)	6 (23)	2 (3)
* cTx*	297 (64)	10 (38)	22 (61)
Clinical N stage			
* cN0*	236 (51)	12 (46)	21 (58)
* cN1*	111 (24)	7 (27)	6 (17)
* cN2*	53 (11)	6 (23)	4 (11)
* cNx*	63 (14)	1 (4)	5 (14)
Clinical M stage			
* cM0*	334 (72)	16 (62)	29 (81)
* cM1*	129 (28)	10 (38)	7 (19)
Pathological T stage			
* (y)pT0*	–	1 (4)	–
* (y)pT1*	–	–	–
* (y)pT2*	14 (3)	1 (4)	3 (8)
* (y)pT3*	268 (57)	16 (62)	24 (67)
* (y)pT4*	180 (39)	8 (31)	9 (25)
* (y)pTx*	1 (0)	–	–
Pathological N stage			
* pN0*	160 (35)	8 (31)	15 (41)
* pN1*	135 (29)	9 (35)	11 (31)
* pN2*	167 (36)	9 (35)	10 (28)
* pNx*	1 (0)	–	–
Pathological M stage			
* pM0*	26 (6)	–	–
* pM1*	40 (9)	8 (31)	5 (14)
* pMx*	397 (85)	18 (69)	31 (86)
Tumour stage (UICC)			
* Stage I*	10 (2)	–	3 (8)
* Stage II*	142 (31)	8 (31)	12 (33)
* Stage III*	244 (53)	10 (38)	16 (44)
* Stage IV*	66 (14)	8 (31)	5 (14)
* Stage X*	1 (0)	–	–
Chemotherapy			
* No chemotherapy*	281 (61)	11 (42)	18 (50)
* Only neo-adjuvant chemotherapy*	0	4 (15)	0
* Only adjuvant chemotherapy*	181 (39)	8 (31)	17 (47)
* Neo- and adjuvant chemotherapy*	1 (0)	3 (12)	1 (3)
90-day mortality^a^	49 (11)	0 (0)	3 (8)

^a^Mortality (90 days) after definite tumour resection

## Discussion

Right-sided OCC was associated with increased 90-day mortality compared to patients without symptoms of obstruction. In addition, after exclusion of patients who died within 90 days, the 5-year OS was also significantly lower in patients with right-sided OCC compared to patients without signs of obstruction. This difference was found in a stratified analysis for all different tumour stages (stage I–II, III and IV). Symptoms of obstruction at the time of diagnosis were independently associated with lower OS in patients with right-sided colon cancer after correction for risk factors such as age, sex, tumour location, pT stage, pN stage, pM stage, and completeness of the resection.

For OCC, high postoperative mortality and morbidity rates have been described previously [22–25]. Emergency surgery for colon cancer has been associated with a significantly increased hospital mortality rate compared to patients without emergency intervention in large population-based studies [26, 27]. Mortality rates after emergency surgery in these studies (8.4% and 10%) were comparable with our results. Alternative treatment strategies to avoid emergency resection, such as stent placement or decompressing stoma, have been mainly investigated in left-sided OCC [4, 15, 16, 28–33]. For right-sided OCC, emergency resection with primary anastomosis or diverting ileostomy is still common surgical practice [1–3, 34]. However, recent studies have shown high morbidity and mortality rates after emergency surgery for right-sided OCC, compared to elective surgery in patient without obstruction, which has been confirmed in this study [7–9, 26, 27, 35].

Even though bridging strategies using stent or stoma have been described for right-sided OCC, these are not widely implemented [2, 5, 11–13, 15, 29]. The present nationwide study confirms that bridging strategies are rarely used in daily practice for right-sided OCC, in contrast to left-sided OCC [12, 36, 37]. However, staged surgery with a bridging strategy using stent or decompressing stoma has shown improvement of short-term postoperative outcome in patients with OCC [1, 3, 12, 17, 38]. In this study, none of the 26 patients receiving bridge to surgery before resection died within 90 days after resection compared to 49 patients (11%) who underwent emergency resection. After excluding patients who died within 90 days in the non-obstructing group 5-year OS was comparable. Although numbers are small with restricted statistical power in the present study, postponing surgery to optimise the preoperative clinical condition might improve short-term outcomes with less postoperative mortality in patients with right-sided OCC and needs further studies to demonstrate the value of this strategy.

The amount of patients treated with emergency surgery for right-sided OCC was slightly different compared to an earlier nationwide study by Amelung et al. This study reported that emergency resection was performed in 95.4% of the patients with right-sided OCC, compared to 88.2% in the present study [1]. Difference in electively treated patients between both studies might be explained by the definition of obstruction, while different databases were used for both studies. In the study of Amelung et al., data was obtained by the Dutch ColoRectal Audit (DCRA) which is filled in by surgeons, compared to IKNL data which is obtained by independent employees. Partial obstruction with a stricture found during colonoscopy might be registered as obstruction even though patients did not experience symptoms of obstruction. Another explanation for the difference in patients treated without emergency resection between both studies may be the use of new treatment strategies. This study showed that 36 patients in the obstruction group were treated electively without the use of stent or decompressing stoma. Clinical management in these patients may have been a relatively new treatment strategy based on tube decompression. In case of an insufficient ileocecal valve, decompression may be accomplished by nasogastric tube placement. Nutrition can be supplemented by total parenteral feeding or low residual enteral feeding (in case of some bowel movement), followed by postponed semi-elective resection. This treatment strategy has not been described widely. However, postponing emergency surgery without using stent or stoma may have been more frequently applied in the present study compared to Amelung et al., who included patients that were treated between 2009 and 2013. Postponing surgery without decompressing stoma or the use of stents has recently been described by Fahim et al. [39]. This study showed that obstruction treatment prevented emergency surgery, seeming to be a safe and efficient alternative to emergency surgery.

Five-year OS, after excluding patients who died within 90 days, was lower in patients with right-sided OCC, compared to patients without obstruction (42% versus 73%), which is comparable with earlier studies [3, 36, 40]. The difference between both groups may be explained by higher surgical stress response combined with worse clinical condition, leading to more postoperative complications compared to elective resection, which in turn leads to longer recovery time [8, 41, 42]. Increased surgical stress response and postoperative complications have both been shown to be associated with worse OS [43, 44]. Furthermore, prolonged recovery in stage III disease may lead to delayed start or even cancellation of adjuvant chemotherapy. Delaying start of adjuvant chemotherapy beyond eight weeks has been significantly associated with worse OS [45]. However, worse survival in case of obstruction was also found in stage I–II disease, not needing adjuvant chemotherapy. The decreased OS found in case of right-sided OCC has been described previously [3]. One study of the French Surgical Association reported a 5-year OS for both left-and right-sided OCC of 43% after excluding postoperative mortality, which is comparable with the present study [2, 3].

The present study has some limitations. This is a retrospective study, analysing patients receiving surgical treatment for right-sided OCC. Patients with stage IV disease, receiving palliative systemic therapy or best supportive care were not included in this study. Therefore, OS in stage IV disease might have been overestimated for both obstructing and non-obstructing right-sided colon cancer. Secondly, different factors influencing mortality and OS in patients with colon cancer, in particular OCC, could not be investigated, e.g. preoperative health status of the patients (BMI, SNAQ score, weight loss), pre- and postoperative complications, bowel decompression strategies during surgery, and postoperative morbidity. Finally, data on recurrences were not available for the total study population, and therefore, the disease-free survival could not be analysed.

## Conclusion

In summary, right-sided OCC was treated with emergency resection in the majority of Dutch patients and led to relatively high postoperative mortality. In addition, a stage-independent worse 5-year OS after excluding 90-day mortality was found in patients with right-sided OCC compared to patients without obstruction. Staged treatment may have the advantage to facilitate elective surgery and provides a chance to optimise the patients’ medical condition. Larger numbers of patients are needed to demonstrate the value of staged treatment in right-sided OCC significantly.

### Supplementary Information

Below is the link to the electronic supplementary material.

Supplementary file1 (DOCX 6473 KB)

## Data Availability

Data can be requested by the corresponding author.
