# Supplementary material for: Surgical treatment and overall survival in patients with right-sided obstructing colon cancer—a nationwide retrospective cohort study
Source: Int J Colorectal Dis. 2023 Oct 5;38(1):248. doi: 10.1007/s00384-023-04541-3 (PMC10556181; doi:10.1007/s00384-023-04541-3)

**Appendix:**

Long-term overall survival obstructing versus non-obstructing right-sided colon cancer. (t0 = date of surgery, postoperative mortality within 90 days were **included** for OS analyses)

**Figure 1a:** Complete group


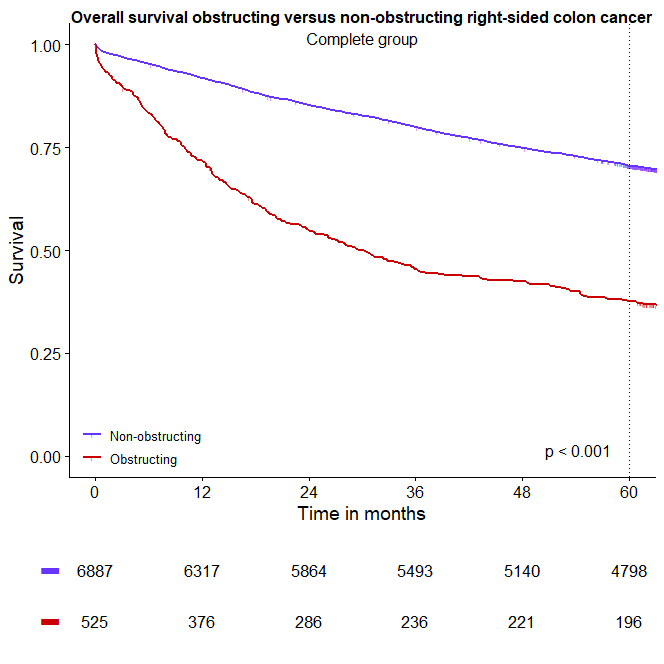


**Figure 1b:** Stadium I-II


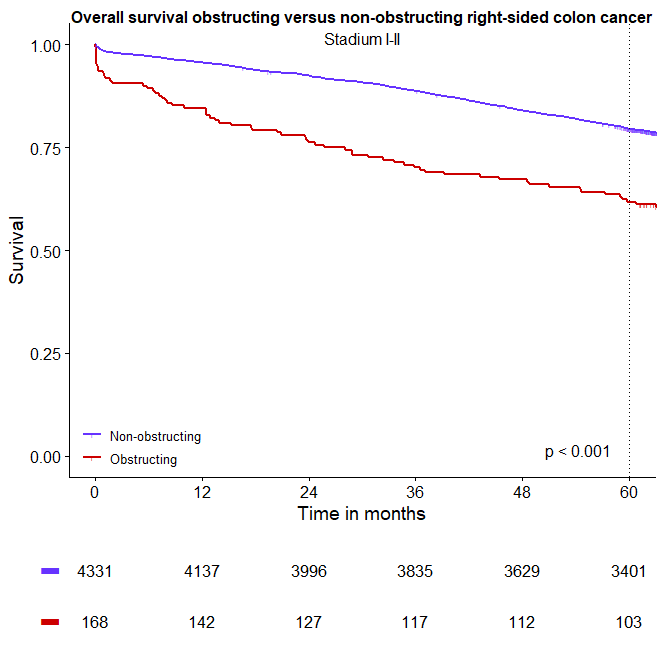


**Figure 1c:** Stadium III


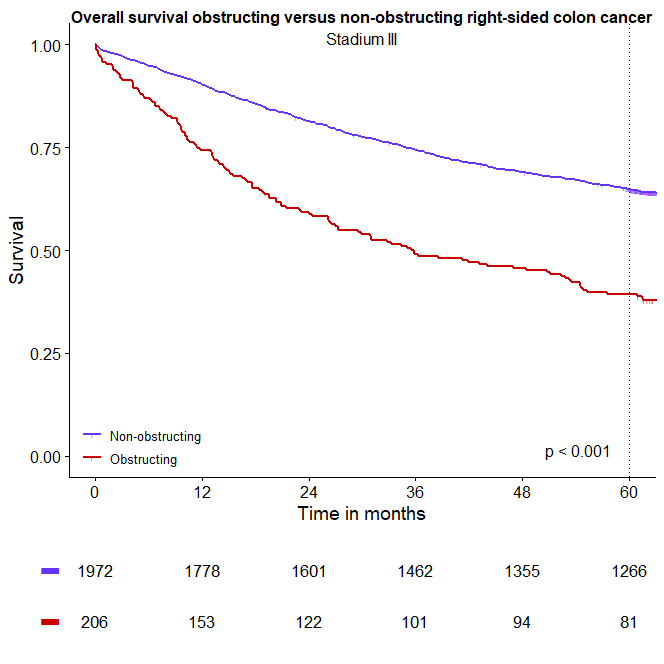


**Figure 1d**: Stadium IV

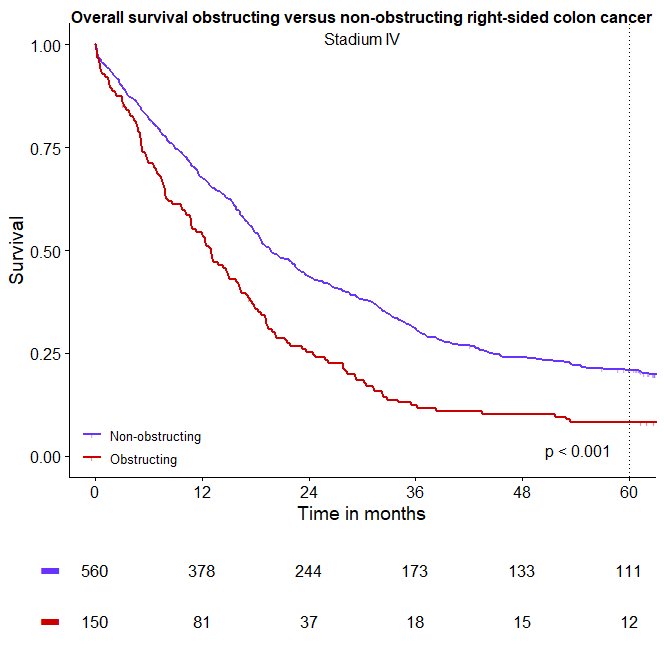


**Figure 1e:** Survival analysis in patients with postoperative mortality < 90 days


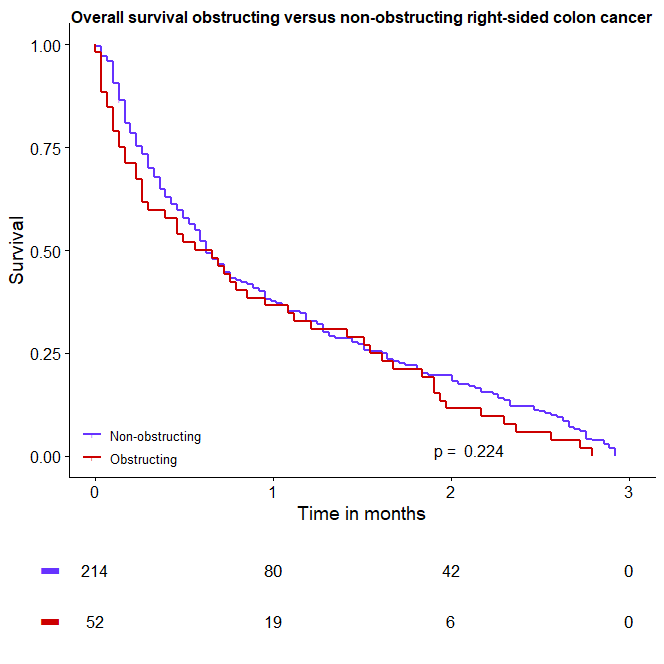

Supplement: Supplementary file 1 — Supplementary file1 (DOCX 6473 KB) [file 384_2023_4541_MOESM1_ESM.docx]
